# A role for genes in the ‘caregiver stress process’?

**DOI:** 10.1038/s41398-018-0275-7

**Published:** 2018-10-22

**Authors:** Douglas A. Wolf, Frank A. Middleton

**Affiliations:** 1 Aging Studies Institute, Syracuse University, Syracuse, NY USA; 2 Department of Neuroscience & Physiology, SUNY Upstate Medical University, Syracuse, NY USA; 3 Department of Psychiatry & Behavioral Sciences, SUNY Upstate Medical University, Syracuse, NY USA; 4 Department of Biochemistry & Molecular Biology, SUNY Upstate Medical University, Syracuse, NY USA

## Abstract

The stress that accompanies caring for one’s parent, and the contribution of that stress to adverse physical and mental-health outcomes, is extensively studied and widely acknowledged. Yet there has been almost no attempt to incorporate the well-documented role of genetic variation in psychological distress into research on caregiving. We use phenotypic data from a large, population-based sample linked to extensive genotype data to develop a polygenic risk score (PRS) for depression, and test for both direct and interactive effects of the PRS in a multilevel repeat-measures model of caregiver-related stress. We distinguish three groups: potential caregivers (those with a living parent who does not need care), noncaregivers (those who do not provide care to their parent that needs care), and caregivers. We also obtain separate estimates according to the gender of both the parent and child. We found that a parent’s need for care, and the child’s provision of care, are associated with depression in some but not all cases; in contrast the PRS was significantly associated with the risk for increased depressive symptoms (with *P* ≤ 0.01) in all cases. These findings support an additive genetic contribution to the diathesis-stress model of depression in the context of caregiving.

## Introduction

Family members, especially adult children, are the primary source of hands-on personal care for older adults that need help with everyday activities such as eating, dressing, bathing, using the toilet, or ambulating indoors^1^. “Parent care” is widely viewed as a burdensome activity, as a source of chronic stress to the care providers, and as a factor contributing to adverse physical and mental health outcomes. Summaries and meta-analyses published over recent decades have repeatedly shown that caregivers have a significantly higher prevalence of psychological distress compared to those not actively providing care^2,3^. Nevertheless, the caregiver literature finds that responses to the caregiving situation are quite heterogeneous: caregiver–noncaregiver differences in outcomes are small or statistically insignificant in many cases, and a number of positive outcomes to the experience of caring have also been reported. Among the factors identified as contributing to this heterogeneity of responses are caregiver attributes such as gender and family situation, the severity of the care recipient’s needs and problem behaviors, external supports and resources available to the caregivers, and the caregiver’s personality traits and coping skills^4,5^.

The social–gerontological literature on caregiver outcomes relies heavily on a “caregiver stress process” model^6,7^, in which depression and anxiety are key outcomes. The focus of this caregiver stress process framework is on those actively involved in care provision, and the empirical literature on this topic uses almost exclusively either caregiver-only samples, or samples of caregivers matched to samples of people not engaged in care provision. Although the theory suggests that the stressors will be experienced, and their consequences manifested, throughout a family network, family members beyond the caregiver-care recipient pair have typically been overlooked. Nevertheless, some research has introduced the distinction between non-caregiving adult children of an parent that actually needs care—that is, “noncaregivers”—and the non-caregiving children of parents that are without care needs, or “potential caregivers.” Some studies have found that the “noncaregiver” group exhibits levels of depressive symptoms that are significantly higher than the “potential caregiver” group, although those differences are in many cases smaller than the differences between active and potential caregivers^8^. Evidence for the existence of “noncaregiver stress” suggests that part of the apparent negative response to the parent-care role is instead a consequence of the parent’s care needs rather than the caregiver’s response to those needs, a response that has been attributed to the child’s exposure to their parent’s suffering^9,10^. Such findings underscore the importance of recognizing all three groups—caregivers, noncaregivers, and potential caregivers—in research that aims to accurately determine parental caregiver consequences using between-group comparisons. In addition, there are well-known gender differences in the need for care^11^, the provision of care^12^, the prevalence of depression^13^, and responses to the caregiver role^14,15^, highlighting the need for differentiating both caregivers and care recipients according to gender.

In addition to those mentioned earlier, genetic background represents another potential source of heterogeneity in the response to the caregiver situation. Yet despite the well-documented role of genetic variation in the neurobiology of stress and stress reactivity^16–18^, there have been few attempts to examine the influence of genetic variation on parent care and its attendant stresses on caregivers. One study, based on a sample of twins, investigated the possibility that shared genetic and environmental factors contribute to the association of caregiving with psychological distress^19^. Comparisons of monozygotic to dizygotic twin pairs revealed that genetic and environmental factors, in combination, underlay the association of caregiving with self-reported measures of mental health functioning, anxiety, and depression, but not of perceived stress. We are aware of only one genetic association study that addressed this issue^20^, which used a small, regional sample (*n* = 288) composed exclusively of caregivers, and considered only a single polymorphism of the serotonin transporter gene (5-HTTLPR).

A general recognition that individuals exposed to similar stressors exhibit a wide range of responses has prompted the development of diathesis-stress theories of depression^21,22^. Only a handful of studies have explicitly tested a diathesis-stress model of caregiver stress responses; in these studies, vulnerability has been variously represented as psychiatric history^23^, baseline physical and mental health^24^, personality traits^5^, and, in the previously cited twins study, genetic and environmental factors^19^.

Here, we use a large, nationally representative sample of active caregivers, noncaregivers, and potential caregivers, in which repeated measures of depressed mood and stressors were collected using structured interviews. Our indicators of a parent’s care needs and their child’s caregiver status are broad, in comparison to the numerous published studies that focus exclusively on a special population such as Alzheimer’s Disease patients and their caregivers. The interview data are linked to an extensive set of high quality genotyping data.

Our goals are fourfold. First, we seek to confirm previously established associations between selected genetic variants and indicators of depressive symptoms, in the specific context of a sample containing both potential and active providers of parent care. From these data, we derive a polygenic risk score (PRS)^25^ in order to define and assess the genetic risk for depressive symptoms in parental caregivers. Second, we investigate whether the variance in depressive symptoms attributable to the PRS scores is large enough to explain previously observed heterogeneity in responses to care-related stressors. Third, we test for interactions between genetic risk and care-related stressors in indicators of depressed mood. Finally, recognizing the importance of gender (of both parent and child) on the caregiver-genes-depression relationships, in all analyses we control for both the parent’s and the child’s gender.

## Materials and methods

### Participants

We used data collected in the Health and Retirement Study (HRS), a longitudinal study funded by the National Institute of Aging and the Social Security Administration that is based primarily on semi-annual interviews of enrolled subjects. The first HRS interviews were conducted in 1992, using a national probability sample of people born 1931–1941 and their spouses. In later years, additional birth cohorts were added to the sample; 1998 was the first year in which the full population of people 50 and older was represented. Additional cohorts were added in 2004 and 2010. The semi-annual interviews cover a broad range of topics including family composition, intergenerational relationships, health and disability, employment, retirement, and economic status. Additional details regarding the design and content of the study are described elsewhere^26^. In the present report, we used data collected in the 1998–2014 interviews to define our analysis sample, measure phenotypes, and to code key stressors and covariates.

Beginning in 2006, subsets of HRS participants consented to the provision of genetic material. In 2006, saliva was collected using a mouthwash collection method, and in 2008, saliva was collected using the Oragene DNA collection kit (OGR-250; DNA Genotek, Ottawa, ON). Saliva completion rates were 83% in 2006 and 84% in 2008. Together the genetic data collected in these two interviews comprise the HRS Genotype Data Version 1, available from dbGaP (accession number phs000428.v1.p1), which we used in this study. Additional information provided through the University of Michigan’s Institute for Survey Research allows researchers to link data from the public-use survey files to these genetic data (https://hrs.isr.umich.edu/data-products/genetic-data). This study was given expedited approval by the Syracuse University Institutional Review Board.

### Genotyping

The NIH Center for Inherited Disease Research (CIDR) used the Illumina Human Omni-2.5 Quad BeadChip (Illumina, San Diego, CA), with coverage of 2.45 million single nucleotide polymorphisms (SNPs), to perform genotyping of the subject DNA samples. Quality control (QC) analysis and data cleaning were performed by the Genetics Coordinating Center at the University of Washington, Seattle, WA^27^. The data release includes files that contain filters for removing records with large chromosomal anomalies, overall missing call rate > 2%, as well as closely related individuals, and removal of SNPs with high missingness and significant violations of Hardy–Weinberg Equilibrium (HWE). The final primary genotyping dataset consisted of 2,201,371 SNPs (after applying the above-mentioned filters) in a total of 12,507 subjects.

In addition to the primary genotypes, an additional 21.6 million SNP genotypes were imputed in study participants by CIDR using a worldwide reference panel of 1092 samples from the 1000 Genomes Project and the IMPUTE2 software^28,29^. Using the genotype probabilities produced by the imputation process, it was possible to calculate a probabilistic dosage code^30^ for the designated allele at each location. The imputation file also included a measure of the quality (certainty) of each imputation. We used PLINK (v1.07)^31^ to extract information on directly genotyped target SNPs in these subjects, coded in dosage form on the forward strand (i.e., 0, 1, or 2 copies of the coded allele). With imputed SNPs, for which probabilistic assignments were provided, the expected dosage (bounded by 0 and 2) is used.

### Samples and measures

We used a two-stage split-sample approach in this study. First, we identified a subsample of HRS participants that had no living parents or parents-in-law during the 1998–2014 study period and who could be linked to the genotype data. These subjects, who did not potentially face parent-care stressors, constituted our development sample (*n* = 4668) and served as the basis for computing a PRS for depressive symptoms. Next, we defined our testing sample, which contained the remaining individuals who on one or more measurement occasions had at least one living parent (*n* = 3203). The testing sample was further stratified by participant gender for the statistical analysis.

Our data were organized into “measurement occasions” that covered two successive interviews, i.e., a 2-year period. This approach was dictated by the wording of key survey questions (as detailed below). Thus, individuals were observed on 1 or more, and up to 8, occasions (1998–2000, …, 2012–2014). Because the spouses or partners of sample individuals were also included as respondents, we further classified participants as single or partnered. By linking participant survey responses to those of their spouses, we were able to include additional covariates (listed below) in our statistical models. To be included in either subsample, participants had to provide complete information on all measured variables for the two successive interviews that comprised each occasion; among couples, we required that the spouse/partner be the same person for those same two interviews.

We considered two key stressors: being a caregiver, and having a parent that needs care. A caregiver variable specific to each parent was coded using responses to questions of the form “Did you spend a total of 100 or more hours in the last two years helping your mother [father] with basic personal activities like dressing, eating, and bathing?” These questions were also asked if the parent in question had died during the 2-year interval. The 100-h threshold filtered out situations of temporary or very low-level caregiving (~1 h per week or less over the 2-year interval). Notably, this measure does not permit us to distinguish short-term high-intensity care episodes from longer-term or continuous care episodes within or throughout the 2-year reference period; moreover, the source data do not include items on the total duration of any care episodes. Accordingly, our indicator of caregiver status refers to those with “recent and/or current” experience as a caregiver. In some analyses we further distinguished between “low-intensity” caregiving (i.e., 1–499 h of care in the last two years) and “high-intensity” caregiving (i.e., 500 or more hours of care in the last 2 years). This 500-h threshold produces a roughly equal classification of caregivers into low-intensity and high-intensity categories.

Care provision, especially for hands-on personal-care tasks such as bathing and dressing, is presumably a response to a disability or chronic condition that creates a need for care. In each biennial interview, HRS participants were asked whether each of their parents was still alive, and whether surviving parents were judged (by the participant or, with specific reference to cognition, by a doctor) to need care according to several specific criteria. Given the 2-year reference period used in the caregiver item, we coded each participant as having a mother or father with care needs if any of three conditions holds: (1) in the current interview, the parent was reported “…to need help with basic personal needs like dressing, eating, or bathing,” or the parent “… cannot be left alone for an hour or more,” or the parent has ever been reported by a doctor to have a “memory-related disease;” (2) any of the 3 preceding specific types of need was reported at the time of the previous interview (i.e., two years before); or (3) the parent died during the 2-year interval between the previous and the current interview. Beginning in 2010, the HRS question about a “memory-related disease” was replaced by the following two questions: “Has a doctor ever told your mother/father that she/he has Alzheimer’s Disease?” and “Has a doctor ever told your mother/father that she/he has dementia, senility or any other serious memory impairment?” We assumed that a positive response to either of the newer items is equivalent to a positive response to the previous version of this question, an assumption supported by supplementary analysis of the reported prevalence of the conditions before and after the wording changes (results not reported). Condition (3) reflects our assumption that in most cases, a parent’s death is preceded by at least a brief period of personal-care needs. Note that in this coding scheme, someone can be coded as a “caregiver” even if they have no living parents at the time they are interviewed; they must, however, have had at least one living parent when interviewed two years in the past. Although we used several criteria for assessing a parent’s need for care, there nevertheless remains some possibility of misclassification. In particular, a surviving parent may have no reported care needs at the time of an interview, and may have had no such needs 2 years in the past, but experienced an episode of disability (e.g., recovery from a fall or hip fracture) that began and ended within the 2-year period, and to which the HRS participant responded by providing personal-care assistance.

The phenotype outcome for this study is depressed mood, which was assessed using 8 yes/no items adapted from the Center for Epidemiologic Studies Depression (CES-D) scale^32^. The CES-D is widely used as a screening device and has been found, in comparison with clinical diagnostic tools, to be effective in detecting both depression and generalized anxiety disorders^33,34^. HRS participants are asked whether “for much of the time during the past week…” they (1) felt depressed; (2) felt that everything they did was an effort; (3) their sleep was restless; (4) they were happy (reverse-coded); (5) felt lonely; (6) enjoyed life (reverse-coded); (7) felt sad; and (8) could not get going. Studies that use these items have often created an additive depressive symptomatology score, ranging from 0 to 8^35–38^. That approach gives equal weight to each of the 8 items, and also overlooks the fact that factors other than depression might underlie some, but not others, of those items (such as sleeplessness). Instead of combining the item scores, we treat the 8 items as multiple indicators of a common latent construct (depressed mood), allowing each to have a unique quantitative association with the underlying construct^8,39,40^. Our approach has the further advantage of permitting inclusion of cases where 1 or more of the 8 depressive symptom items are missing; otherwise, we would need to either delete the partially missing case (which discards some data) or impute the additive score (which introduces additional uncertainty regarding parameter estimates), either of which alternative approaches would require (like ours) a missing-at-random assumption. Further evidence to support our decision to treat the 8 CES-D items as separate indicators is included in Supplementary Table 1.

In addition to the preceding variables, we control for several previously studied stressful life events, such as the death of a spouse or partner in the last 2 years or the recent loss of a job^37,40^, or, among those with a spouse, the spouse’s job loss^41^. Other established correlates of depressed mood include self-reported disabilities with activities of daily living (ADLs: eating, dressing, bathing, mobility, transfer, and toileting)^42^, the presence of a spouse or partner^43^, and the count of spouse’s ADL disabilities^44^. Additional covariates include whether the parent is married, the participants’ age, their numbers of sisters, brothers, and children and, if partnered, their number of sisters-in-law and brothers-in-law, indicators of race/ethnicity (Black; Hispanic), and indicators of the calendar year in which the CES-D items were collected. Indicators of a spouse’s job loss, ADL count, and counts of brothers and sisters came from the linked spouse interviews. We also included the first four eigenvalues from a principal components analysis (PCA) of the genetic data designed to detect and enable controlling for population stratification; preliminary analyses indicated that additional eigenvalues did not add to the explanatory power of the model. All of the variables mentioned in this paragraph are treated as covariates.

### Statistical analysis

Participants appear in the analysis on one or more measurement occasions and provide up to eight binary indicators of depression at each occasion. This creates a multilevel repeated-measures situation, for which we use a random-effects logistic regression model:

1 $$\begin{array}{l}{\mathop{\rm I}\nolimits} {\mathrm{n}}\left[ {{\mathrm{pr}}\left( {y_{ijt} = 1} \right)/1 - {\mathrm{pr}}\left( {y_{ijl} = 1} \right)} \right] = \beta _{0j} + \beta _1N_{it} + \beta _2C_{it}\\ + \beta _3G_i + \beta _4X_{it} + \theta _i + \varepsilon _{it},\end{array}$$

for individual *i*, measurement occasion *t*, and depression indicator *j*. In (1), *N* refers to the parent’s need for care, *C* for the child’s provision of care, *G* is the Polygenic Risk Score (see next section), and *X* represents additional controls. The need (*N*) and care (*C*) variables appear separately for the participant’s mother and father, as applicable. As presented, this model assumes that need and caregiving have additive effects; this assumption can be tested by adding the multiplicative effect *N* × *C*. Similarly, a mediating (in addition to the hypothesized direct) effect of genes on depression among caregivers can be tested by inclusion of the multiplicative effects *N* × *G* and *C* × *G*. All models were estimated using the “melogit” routine in Stata (version 14).

### Development of polygenic risk score for depression

We began with a list of 106 preselected SNPs (Supplementary Table 2), all of which are contained in the HRS genetics data release, and each of which has been found in one or more published studies to have a significant direct, additive association with some measure of mental health, most often an indicator of depression such as the CES-D, and in some cases a diagnosis of major depressive disorder or post-traumatic stress disorder. A few of the SNPs on this list came from association studies of related outcomes such as stress reactivity, coping styles, or anxiety disorders. We also included two SNPs that have been found to form a haplotype that is closely related with the repeat polymorphism 5-HTTLPR^45^, which has, in turn, been found to predict depressive disorders in numerous studies^46^. We tested for both main and interactive effects of these two SNPs.

Using the development sample previously described, we tested for an association between each of these 106 SNPs and depressed mood using Eq. (1), excluding *N* and *C*, as they are undefined in this group. For 13 SNPs, a nominal *P*-value ≤ 0.05 was obtained; one of these was dropped because it was in complete linkage disequilibrium with another (*R*^2^ = 1). Further controlling for false discoveries using a *q*-value of 0.10^47^ left us with a list of four SNPs with which to create the PRS.

The four SNPs retained for use in calculating the PRS are listed in Table 1, along with the gene (or intergenic region) and annotation, where known. One of the 4 SNPs was imputed rather than directly assayed. All of these SNPs were previously associated with an 8-item CES-D score in two separate studies based on the same HRS data as we are using, albeit for somewhat different years, subsamples, and with different controls for covariates^35,36^. To address our initial goal, we then calculated a weighted PRS for each subject in the testing sample, with weights equal to the logistic regression coefficients estimated using the development sample.

**Table 1:** SNPs included in polygenic risk score (PRS)

rsID	rs6552764	rs7002316	rs12921740	rs58682566^a^
Chr	4	8	16	18
Gene	ENPP6	LOC105375856	Intergenic	EPG5
Source	Ware et al. ^23^	Levine et al.^22^	Ware et al.^23^	Levine et al.^22^
Position	184183350	57904885	20300710	45951902
Alleles	A/T	A/C	A/C/T	A/G
1KG MAF	*T* = 0.1707	*C* = 0.4020	*C* = 0.2502	*G* = 0.1753
Variant Class	intron	Intron	Regulatory	Intron
Functional Annotation	Ectonucleotide pyrophosphatase/phosphodiesterase 6; Hydrolyzes *p*-nitrophenyl phosphorylcholine (pNPPC), *p*-nitrophenyl phenylphosphate (pNPPP), sphingosyl-phosphorylcholine and platelet-activating factor (PAF).	Non-coding RNA	CFTF transcription factor binding site	Ectopic P-granules autophagy protein 5 homolog; Involved in catabolic autophagy, endosome recycling, autophagosome maturation
PRS weight	0.1885	0.1219	0.1042	0.2514

^a^Imputed

## Results

Selected characteristics of the development and the two gender-stratified testing samples are shown in Table 2. The development sample is considerably older than the testing samples, which is to be expected given that individuals in the development sample have no living parents, while those in the testing samples do. Given these age differences, the prevalence of spousal death is higher, while the prevalence of job loss is lower, in the development sample relative to the testing samples.

**Table 2:** Selected characteristics of analysis samples

Characteristic^a^	Development sample	Testing samples	
		Daughters	Sons
Age (mean ± sd)	74.0 ± 8.9	62.2 ± 5.8	61.9 ± 5.6
Mother needs care (%)	^b^	44.9	43.2
Child provides care to mother (%)	^b^	14.6	6.6
Low-intensity care (%)	^b^	6.8	3.9
High-intensity care (%)	^b^	7.8	2.7
Father needs care (%)	^b^	14.9	15.3
Child provides care to father (%)	^b^	3.4	2.3
Low-intensity care (%)	^b^	1.7	1.5
High-intensity care (%)	^b^	1.7	0.8
PRS (mean ± sd)	^c^	0.55 ± 0.20	0.56 ± 0.20
PRS missing (%)	^c^	1.5	1.9
Spouse died (%)	3.5	1.5	0.6
Job loss (%)	1.8	3.0	4.3
ADL disability count (mean ± sd)	0.26 ± 0.73	0.19 ± 0.66	0.12 ± 0.52
Has spouse (%)	49.8	61.7	81.0
Spouse’s job loss (%)	0.8	2.5	2.0
Spouse’s ADL disability count (mean ± sd)	0.12 ± 0.54	0.11 ± 0.52	0.12 ± 0.52
Number of individuals	4668	1712	1491
Number of occasions	29967	6470	5497

*PRS* polygenic risk score, *ADL* activities of daily living

^a^All sample characteristics computed using data pooled over all measurement occasions

^b^Individuals in development sample have no living parents

^c^PRS not computed in development sample

Table 2 reveals higher levels of needs among mothers than fathers, a greater tendency of the children to provide care for their mothers than for their fathers, and a substantially greater prevalence of care provision by daughters than by sons. In contrast, the sons are much more likely than the daughters to have a spouse present, reflecting gender differences in marriage or remarriage patterns and survivorship. Together these features illustrate the importance of stratifying the analysis by both the parent’s gender and the child’s gender. The experience of a spouse’s death, loss of a job, or own disability are all relatively rare in these groups.

We used the PRS described above, along with the binary indicators of each parent’s need for care (*N*) and the participant’s provision of care for each parent (*C*) and the previously described covariates, in a random-effects multiple-indicator logistic regression (Eq. [1]). Given the stratification by the child’s gender and the separate controls for mothers’ and fathers’ care needs and caregiving, we conducted four tests of the hypothesized associations. The PRS (*G*) in these models is centered at its sample mean. With modest losses due to missing values (1.5% among daughters and 1.9% among sons), sample sizes are 1712 daughters and 1491 sons. Each was observed on multiple occasions, providing up to eight depression indicators on each occasion, so the number of data points included in each estimation is much larger.

Results for our basic main-effects models are shown in panel (a) of Tables 3, 4. Both of the PRS main effects are statistically significant with *P*-values of 0.01 or less. The genetic effect is slightly larger for sons than for daughters. Thus, for the first of our four questions—does genetic variation contribute to depressive symptom outcomes among individuals differentiated according to actual and potential caregiver status?—the answer is an unqualified yes. The standard finding in the literature—namely, an adverse psychological response to being a caregiver—appears only once, among mother–daughter pairs. “Noncaregiver stress”—an adverse psychological response to one’s parent’s needs for care—is found among mother–daughter, father–daughter, and mother–son pairs. Note also that among mother–daughter pairs, the only case to exhibit both caregiver and noncaregiver stress, the caregiver stress response is only trivially larger than the noncaregiver stress response.

**Table 3:** Effects of genes and stressors on depressed mood, daughters

Variable	*β*	SE	*P*	LCI	UCI
(a)					
N—mother	0.2448	0.0695	<0.001	0.1087	0.3810
C—mother	0.2910	0.0840	0.001	0.1263	0.4557
N—father	0.2618	0.0968	0.007	0.0721	0.4515
C—father	0.2970	0.1606	0.064	−0.0177	0.6118
G	0.7153	0.2761	0.010	0.1741	1.2565
Spouse died	0.9237	0.1898	<0.001	0.5517	1.2958
Job loss	0.0789	0.1520	0.604	−0.2191	0.3769
ADL disability count	0.2996	0.0451	<0.001	0.2113	0.3880
Has spouse	−0.6498	0.1157	<0.001	−0.8766	−0.4230
Spouse’s job loss	0.3188	0.1721	0.064	−0.0185	0.6560
Spouse’s ADL disability count	0.1191	0.0610	0.051	−0.0004	0.2386
(b)					
N—mother	0.2403	0.0696	0.001	0.1040	0.3767
C—mother—low intensity	0.2047	0.1074	0.057	−0.0059	0.4153
C—mother—high intensity	0.3794	0.1081	<0.001	0.1675	0.5913
N—father	0.2615	0.0969	0.007	0.0717	0.4514
C—father—low intensity	0.2924	0.2022	0.148	−0.1040	0.6887
C—father—high intensity	0.3039	0.2229	0.173	−0.1329	0.7408
G	0.7204	0.2761	0.009	0.1792	1.2615
Number of individuals			1686		
Number of occasions			6370		
Number of indicators			50,923		

*N*, parent’s need for care, *C* child’s provision of care, *G* genetic risk score, *ADL* activities of daily living, *P* *P*-value from two-sided *t*-test, *LCI (UCI)* lower (upper) limits of 95% confidence interval for *β*

**Table 4:** Effects of genes and stressors on depressed mood, sons

Variable	*β*	SE	*P*	LCI	UCI
(a)					
N—mother	0.3539	0.0743	<0.001	0.2082	0.4996
C—mother	−0.0130	0.1272	0.919	−0.2622	0.2362
N—father	0.0073	0.1060	0.945	−0.2005	0.2151
C—father	0.3215	0.2130	0.131	−0.0960	0.7390
G	0.8974	0.3102	0.004	0.2894	1.5053
Spouse died	1.0029	0.3114	0.001	0.3927	1.6132
Job loss	0.2300	0.1403	0.101	−0.0449	0.5049
ADL disability count	0.3510	0.0562	<0.001	0.2409	0.4612
Has spouse	−0.7587	0.1313	<0.001	−1.0161	−0.5014
Spouse’s job loss	−0.3212	0.2171	0.139	−0.7467	0.1042
Spouse’s ADL disability count	0.1523	0.0611	0.013	0.0326	0.2720
(b)					
N—mother	0.3534	0.0743	<0.001	0.2077	0.4991
C—mother—low intensity	−0.0340	0.1567	0.828	−0.3411	0.2731
C—mother—high intensity	0.0212	0.1843	0.908	−0.3401	0.3825
N—father	0.0054	0.1062	0.960	−0.2028	0.2135
C—father—low intensity	0.2657	0.2604	0.308	−0.2447	0.7761
C—father—high intensity	0.4197	0.3335	0.208	−0.2339	1.0733
G	0.8973	0.3102	0.004	0.2893	1.5052
Number of individuals			1463		
Number of occasions			5393		
Number of indicators			43,121		

*N*, parent’s need for care, *C* child’s provision of care, *G* genetic risk score, *ADL* activities of daily living, *P* *P*-value from two-sided *t*-test, *LCI (UCI)* lower (upper) limits of 95% confidence interval for *β*

These main-effects models assume additive effects of the three key variables, *N*, *C*, and *G*. We tested for non-additive effects in the stress process part of the model by adding *N* × *C* terms. In no case was the interaction significant, lending support to the initial assumption of additivity. We also tested for a moderating effect of the PRS on depression, adding two interaction effects (*N* × *G* and *C* × *G*). None of these interaction variables was individually significant; nor could we reject the null hypothesis that neither variable belongs in the model, based on a likelihood-ratio test. Thus, for the third of our four research questions—do genes moderate the caregiver stress process?—we conclude that the answer is no.

To address whether genetic factors could explain the heterogeneous responses to caregiving widely acknowledged in the social–gerontological literature, we compared the size of the *G* effect to the *N* and *C* effects found among mother–daughter pairs, the only group for which both the *N* and the *C* effects are significantly different from zero. Given the evidence in support of additive effects, the total effect on depression for a caregiving daughter is the sum of these two effects, that is 0.2448 + 0.2910 = 0.5358. For purposes of comparison to the effects of genetic variation, we examined the cumulative distribution of the PRS, weighted by effect magnitude (0.7153). We found that the difference between roughly the 5th and the 95th percentiles of the effect-weighted PRS distribution was as large as the combined effects of the mother’s need for care and the daughter’s provision of care. Thus, only small subsets of the population (about 10%) are different enough with respect to their genotypes that they can be expected to exhibit differences in depression scores equivalent to those of caregivers, compared to the children of parents without care needs. Consequently, we conclude that genetic differences in the population have effects on depressive symptoms that are comparable to, but not notably larger than, the effects associated with caregiver stress.

Some context for these genetic and care-related depression outcomes is provided by the additional regression results shown in Tables 3, 4. In particular, men and women who have experienced a spouse’s recent death have much higher depression scores than those who have not. Both models reveal depression levels that are markedly lower among individuals with a living spouse than among those that are single. Notably, we also found that a one-unit increase in the number of one’s own ADL disabilities (on a 0─5 scale) is associated with an increase in depression comparable to that of having a parent with care needs.

Results contrasting low-intensity and high-intensity caregivers are shown in panel (b) of Tables 3, 4. Although the point estimates suggest that high-intensity caregiving raises the depression score somewhat more than does low-intensity caregiving, as we have classified them, only the mother–daughter result achieves statistical significance.

We explored additional dimensions of the caregiver stress process in supplementary analyses. First, we pooled the samples of daughters and sons in order to obtain a direct estimate of gender differences in depressed mood. A main-effects model (Supplementary Table 3) confirms that depression scores in females are considerably higher than males, after accounting for both parents’ needs for care and the children’s provision of care. However, a likelihood-ratio test rejects this pooled model in favor of the stratified models reported in Tables 3, 4 (*P* < 0.0001).

Finally, we tested the sensitivity of results to the inclusion of recently deceased parents in the “need for care” category (Supplementary Table 4). The overall pattern of findings is unchanged in these models, although SEs are somewhat larger due to the reduction in sample size. In particular, all of the statistically significant results (for *N*, *C*, and *G*) reported in Tables 3, 4 are preserved.

## Discussion

Our analysis reveals several sources of heterogeneity in the depressive symptoms manifested among family caregivers, including the gender of the caregiver, the gender of the care recipient, and (for women) the time intensity of their caregiving activity. The strongly gendered nature of caregiver behavior and its consequences are well-documented, and have been attributed to numerous factors. In addition to the demographic characteristics that we have controlled for—a greater prevalence of widowhood and care needs among mothers than among fathers—the socialization of women into a caring role^48^, the uniqueness of mother–daughter relationships^49^, and a preference, among both men and women, for same-gender caregivers^50^, have all been noted.

Genetic variation also contributes to heterogeneity in depressive symptoms among caregivers. For some combinations of parent’s and child’s gender we find no adverse consequences of the parents’ needs for care, and for some there are no adverse consequences of providing care. However, for all groups there is a significant genetic contribution to depression. Notably, among daughters, underlying differences in genetic predisposition to depression are larger than the combined effects of *N* and *C*, comparing women in the lower and upper tails of the *G* distribution.

Both additive and interactive varieties of diathesis-stress models of depression have been proposed^22^, and support for both forms of this model can be found in the literature^37,40^. Most empirical tests of this model focus exclusively on acute stressors, generally in the form of major life events^51^. Our analysis considers hands-on provision of care to one’s parent, which for most caregivers is a response to a chronic condition rather than a discrete life event. Our results support an additive version of the diathesis-stress model of depression; we find no evidence for an interaction between genetic vulnerability and care-related stressors.

We acknowledge several limitations of our analysis. While parent care is generally characterized as a chronic stressor, we have no direct measure of chronicity. Furthermore, our measure of a parent’s need for care includes cases of a parent that has recently died, an occurrence often counted as a stressful life event (i.e., an acute stressor). Nevertheless, eliminating the cases with recently deceased parents did not change the findings (Supplementary Table 4). As in many other studies of caregiver distress, our measures of the parents’ care needs and their children’s caregiver outcomes rely on self-reported survey responses. However, in contrast to those studies that use a clinically diagnosed or practice-based sample, we are able to test our models using a much larger sample, randomly selected from the general population, and representing a broad range of care needs. Finally, a child’s decision regarding whether, and how intensively, to provide parent care is a matter of choice, and there may be unmeasured factors implicated in both the caregiver decision and depression-proneness, such as personality traits, motivations, or coping skills. These unmeasured variables could, if present, produce bias in our estimates of the effects of caregiving on depression; this unmeasured-variables argument, however, is less likely to pertain to the need-for-care findings.

Our findings have implications for clinical practice and programmatic interventions. Given that nearly half of the depression found among daughters caring for their mothers results from the mothers’ care needs rather than the daughters’ caregiving, efforts to reduce the daughters’ depression through the provision of supportive or respite services are inherently limited. Moreover, programs focused solely on active caregivers fail to address the mental-health consequences exhibited among the non-caregiving family members.

The potential neurobiological implications of our major findings also bear brief discussion. By far, the largest effect that we detected among the 106 tested SNPs was for a somewhat uncommon variant (rs58682566; MAF = 0.1753) located in the intron of EPG5. This gene encodes Ectopic P-granules autophagy protein 5, which functions as a Rab7 effector that has a major role in regulating autophagosome fusion with late endosomes or lysosomes. A recent study identified this same SNP as the single genome-wide significant finding in a discovery cohort of subjects with MDD^52^. Thus, the EPG5 SNP with the largest effect size in the present study gains considerable support for its important in other recent studies of MDD. Although the functional implications of this particular intronic SNP are difficult to predict, there is also strong support for a low tolerance of EPG5 sequence variants in normal health and aging. For example, mutations in EPG5 are associated with Vici syndrome, a multisystem disorder with defective autophagy, agenesis of the corpus callosum, neurodevelopmental delay and neurodegeneration (Online Mendelian Inheritance in Man entry OMIM242840). Seven EPG5 SNPs were also recently associated with the risk of Alzheimer’s disease and another eight associated with the age of onset of Alzheimer’s disease^53^.

These findings on EPG5 are bolstered by the considerable evidence associating abnormal or defective autophagy in various neurodegenerative diseases, including AD, Parkinson’s disease, amyotrophic lateral sclerosis, and others (reviewed by Jia and Le^54^). Of relevance to the current study, defective autophagy has been proposed as a key element in the pathogenesis of MDD and the effectiveness of anti-depressant therapy has been proposed to depend on upregulation of autophagy^54^. Similarly, the upregulation of autophagy was recently proposed to underlie the anxiolytic and anti-depressive effects of nicotine in a mouse model of chronic unpredictable mild stress^55^. Combined with our present findings, these collective observations suggest that the physiological effects of caregiver stress may be mediated in part by sequence variants in EPG5 that directly alter autophagy, thus forming a means for psychological stress to translate into cellular stress.

In conclusion, we have identified a strong genetic contribution to the risk for depression in a longitudinal cohort of adults who are actively providing care for their parents. The magnitude of the genetic effect as determined through a polygenic risk score derived from an independent training sample was determined to be equal to, but largely independent from, other environmental, psychosocial, and care-related variables in the testing sample. Future studies are clearly indicated to explore the implications of our findings in more detail and uncover potential biological mechanisms. Some of these studies could focus on the specific genes and variants that were highlighted in this study in other large cohorts of adults at risk for depression as a result of caregiver stress. Prospective screening of new parental or general caregivers of older adults using the PRS score might also help to target individuals at risk of depressive outcomes.

## Electronic supplementary material

supp table 2 part 2

supp table 1

supp table 2 part 1

supp table 3

supp table 4
