# Supplementary material for: A role for genes in the ‘caregiver stress process’?
Source: Transl Psychiatry. 2018 Oct 22;8:228. doi: 10.1038/s41398-018-0275-7 (PMC6197190; doi:10.1038/s41398-018-0275-7)
Supplement: Supplementary file 1 — supp table 2 part 2 [file 41398_2018_275_MOESM1_ESM.docx]

Sources listed in Supplementary Table 2:

1. Hyde CL, Nagle MW, Tian C, Chen X, Paciga SA, Wendland JR, et al. Identification of 15 genetic loci associated with risk of major depression in individuals of European descent. *Nat Genet* 2016; 48: 1031-1036.
2. Levine ME, Crimmins EM, Prescott CA, Phillips D, Arpawong TE, Lee J. A polygenic risk score associated with measures of depressive symptoms among older adults. *Biodemogr Soc Biol* 2014; 60: 199-211.
3. Ware EB, Mukherjee B, Sun YV, Diez-Roux AV, Kardia SLR, Smith JA. Comparative genome-wide association studies of a depressive symptom phenotype in a repeated measures setting by race/ethnicity in the multi-ethnic study of atherosclerosis. *BMC Genet* 2015; 16:118.
4. Pearson-Fuhrhop KM, Dunn EC, Mortero S, Devan WJ, Falcone GJ, Lee P, et al. Dopamine genetic risk score predicts depressive symptoms in healthy adults and adults with depression. *PLOS One* 2014; 9(5): e93772.
5. Rodrigues SM, Saslow LR, Garcia N, John OP, Keltner D. Oxytocin receptor genetic variation relates to empathy and stress reactivity in humans. *P Natl Acad Sci USA* 2009; 106: 21437-21441.
6. McMahon FJ, Akula N, Schulze TG, Muglia P, Tozzi F, Detera-Wadleigh SD, et al. Meta-analysis of genome-wide association data identifies a risk locus for major mood disorders on 3p21.1 *Nat Genet* 2010; 42: 128-131.
7. Demirkan A, Lahti J, Direk N, Viktorin A, Lunetta KL, Terracciano A, et al. Somatic, positive and negative domains of the Center for Epidemiological Studies Depression (CES-D) scale: A meta-analysis of genome-wide association studies. *Psychol Med* 2016; 46: 1613-1623.
8. Stein MB, Chen C-Y, Ursano, Cai T, Gelernter J, Heeringa SG, et al. Genome-wide association studies of posttraumatic stress disorder in 2 cohorts of US Army soldiers. *JAMA Psychiat* 2016; 73: 695-704.
9. Szczepankiewicz S, Leszczyńska-Rodziewica A, Pawlak J, Narozna B, Rajewska-Rager A, Wilkosc M, et al. PKBP5 polymorphism is associated with major depression but not with bipolar disorder. *J Affect Disorders* 2014; 164: 33-37.
10. Pinsonneault JK, Sullivan D, Sadee W, Soares CN, Hampson E, Steiner M. Association study of the estrogen receptor gene ESR1 with postpartum depression—a pilot study. *Arch Women’s Ment Health* 2014; 16: 499-509.
11. Binder EB, Bradley RG, Liu W, Epstein MP, Deveau TC, Mercer KB, et al. Association of FKBP5 polymorphisms and childhood abuse with risk of posttraumatic stress disorder symptoms in adults. *J Amer Med Assoc* 2008; 299: 1291-1305.
12. Velders FP, Kuningas M, Kumari M, Dekker MJ, Uitterlinden AG, Kirschbaum C, et al. Genetics of cortisol secretion and depressive symptoms: A candidate gene and genome wide association approach. *Psychoneruoendrocrino* 2011; 36: 1053-61.
13. Ressler KJ, Mercer KB, Bradley B, Jovanovic T, Mahan A, Kerley K, et al. Post-traumatic stress disorder is associated with PACAP and the PACI receptor. *Nature* 2011; 470: 492-297.
14. Roetker NS, Yonker JA, Lee C, Chang V, Basson JJ, Roan CL, et al. Multigene interactions and the prediction of depression in the Wisconsin Longitudinal Study. *BMJ Open* 2012; 2: e000944.
15. Demirkan A, Penninx BWJH, Hek K, Wray NR, Amin N, Aulchenko YS, et al. Genetic risk profiles for depression and anxiety in adult and elderly cohorts. *Mol Psychiatr* 2011; 16: 773-783.
16. Hek K, Mulder CL, Luijendijk HJ, vanDuijn CM, Hofman A, Uitterlinden AG, et al. The PCLO gene and depressive disorders: replication in a population-based study. *Hum Mol Genet* 2010; 19: 731-4.
17. Hines LM, Hoffman PL, Bhave S, Saba L, Kaiser A, Snell L, et al. A sex-specific role of type VII adenyl cyclase in depression. *J Neurosci* 2006; 26: 12609-12619.
18. Aizawa S, Ishitobi Y, Masuda K, Inoue A, Oshita H, Hirakawa H, et al. Genetic association of the transcription of neuroplasticity-related genes and variation in stress-coping style. *Brain Behav* 2015; 5(9), e00360.
19. Avoshina V, Mocchetti H, Liu C, Young MA, Anastos K, Cohen M, et al. Single nucleotide polymorphisms in TrkB and risk for depression: Findings from the women’s interagency HIV study. *JAIDS* 2013; 64: 138-141.
20. Logue MW, Solovieff N, Leussis MP, Wolf EK. Melista E, Baldwin C, et al. The ankyin-3 gene is associated with posttraumatic stress disorder and externalizing comorbidity. *Psychoneuroendrocrino* 2013; 38: 2249-57.
21. van Winkel M, Peeters F, van Winkel R, Kenis G, Collip D, Geschwind N, et al. Impact of variation in the BNDF gene on social stress sensitivity and the buffering impact of positive emotions: replication and extension of a gene-environment interaction. *Eur Neuropsychopharm* 2014; 24: 930-938.
22. Nyman ES, Sulkava S, Soronen P, Miettunen J, Loukola A, Leppä V, et al. Interaction of early environment, gender and genes of monoamine neurotransmission in the aetiology of depression in a large population-based Finnish birth cohort. *BMJ Open* 2011; 1(1): e000087.
23. Gizatullin R, Zaboli G, Jönsson EG, Åsberg M, Leopardi R. Haplotype analysis reveals tryptophan hydroxylase (TPH) 1 gene variants associated with major depression. *Biol Psychiat* 2006; 59: 295-300.
24. Kravitz HM, Janssen I, Lotrich FE, Kado DM, Bromberger JT. Sex steroid hormone gene polymorphisms and depressive symptoms in women in midlife. *Am J Med* 2006; 119 (9 supp): S87-93.
25. Klinedinst NJ, Resnick B, Yerges-Armstrong LM, Dorsey SG. The interplay of genetics, behavior, and pain with depressive symptoms in the elderly. *Gerontologist* 2015; 55 (S1): S67-77.
26. Pan Y, Cheng Q, Shan M-S, Yan J. Association between polymorphism of the norepinephrine transporter gene rs2242446 and rs5669 loci and depression disorders. *Int J Clin Exp Med* 2015; 8: 18837-18842.
27. Marques FZ, Eikelis N, Bayles RG, Lambert EA, Straznicky NE, Hering D, et al. A polymorphism in the norepinephrine transporter gene is associated with affective and cardiovascular disease through a microRNA nechanism. *Mol Psychiatr* 2017; 22: 134-141.
28. Heck A, Lieb R, Eligas A, Pfister H, Lucae S, Erhardt A, et al. Polymorphisms in the angiotensin-converting enzyme gene region predict coping styles in health adults and depressed patients. *Am J Med Genet B* 2008; 150B: 104-114.
29. Arpawong TE, Lee J, Phillips DF, Crimmins EM, Levine ME, Prescott CA. Effects of recent stress and variation in the serotonin transporter polymorphism (5-HTTLPR) on depressive symptoms: A repeated-measures study of adults age 50 and older. *Behav Genet* 2016; 46: 72-88.
30. Nikolova Y, Bogdan R, Pizzagalli DA. Perception of a naturalistic stressor interacts with 5-HTTLPR/rs25531 genotype and gender to impact reward responsiveness. *Neuropsychobiology* 2012; 65: 45-54.
31. Jonas W, Mileva-Seitz V, Girard AW, Bisceglia R, Kennedy JL, Sokolowski M, et al. Genetic variation in oxytocin rs2740210 and early adversity associated with postpartum depression and breastfeeding duration. *Genes Brain Behav* 2013; 12(7): 681-94.
32. Funke B, Malhotra AK, Finn CT, Plocik AM, Lake SL, Lencz T, et al. COMT genetic variation confers risk for psychotic and affective disorders: a case control study. *Behav Brain Funct* 2005; Oct 18; 1:19.
33. Stein MB, Fallin MD, Schork NJ, Gelernter J. COMT polymorphisms and anxiety-related personality traits. *Neuropsychopharmacolo* 2005; 30: 2092-2102.
34. Brummett BH, Babyak MA, Williams RB, Harris KM, Jiang R, Kraus WE, et al. A putatively functional polymorphism in the HTR2C gene is associated with depressive symptoms in white females reporting significant life stress. *PLOS One* 2014; 9(12): e114451.
